# Public and Private Information Sharing under “New Normal” of COVID-19: Understanding the Roles of Habit and Outcome Expectation

**DOI:** 10.3390/ijerph19095552

**Published:** 2022-05-03

**Authors:** Han Lv, Xueyan Cao, Shiqi Chen, Liqun Liu

**Affiliations:** 1 School of Journalism and Communication, Wuhan University, Wuhan 430072, China; lvhan3322@126.com (H.L.); caoxueyanluojia@whu.edu.cn (X.C.); 2 Sichuan Academy of Social Sciences, Chengdu 610071, China; irischen47@foxmail.com; 3 National Institute of Cultural Development, Wuhan University, Wuhan 430072, China; 4 Center for Studies of Media Development, Wuhan University, Wuhan 430072, China

**Keywords:** information sharing, habit, outcome expectations, private sharing, public sharing

## Abstract

Information sharing is critical in risk communication and management during the COVID-19 epidemic, and information sharing has been a part of individual prevention and particular lifestyles under the “New Normal” of COVID-19. Thus, the purpose of this study was to explore influencing factors and mechanisms in public and private information sharing intention among people under the regular risk situation. This study investigated an information sharing mechanism based on a cross-sectional design. We collected 780 valid responses through a sample database of an online questionnaire platform and utilized partial least squares structural equation modeling (PLS-SEM) to further analyze the data. To explore the difference caused by news frames, we divided respondents into two groups according to the news frame (action frame vs. reassurance frame) and proceeded with the multi-group analysis. The results showed that four types of outcome expectations (information seeking, emotion regulation, altruism and public engagement) and habit had impacts on public and private information sharing intention. Two paths influencing information sharing proposed in this study were supported. The results showed that outcome expectations were positively related to habit, which implies that the cognitive mechanism was positively relevant to the formation of habit. The results proved that habit played a mediating role between outcome expectations and information sharing. This research found that emotion regulation and public engagement outcome expectations only affected two types of information sharing intention mediated by habit. Regarding the role of the news frame, this study found no significant difference between the group exposed to action-framed news and the group exposed to reassurance-framed news. By exploring influencing factors and the mechanism of information sharing under the “New Normal”, these findings contribute to understanding of information sharing and have implications on risk management. The proposed mechanism classifying public and private information sharing complements risk information flowing by considering online risk incubation.

## 1. Introduction

Information diffusion is an essential component in communication and plays a role in public issues. First, information exchange can contribute to individual and social long-term development. The evidence shows that health information in interpersonal communication, online communities, and professional guidance is increasingly significant [1,2]. Second, communication in risk events has been viewed primarily as disseminating information to the public about emergencies. According to World Health Organization, risk communication can contribute to encouraging informed decision making, positive behavior change, and maintenance of trust [3]. Reddy and Gupta suggested that empowering people with the correct information was the most crucial factor in preventing the spread of the virus [4].Conversely, Cinelli et al. have shown concerns about the negative effect of widely-spread misinformation [5]. In terms of the risk information diffusion process, social media platforms provide spaces to exchange information freely with more significant amounts of people in a lesser amount of time than traditional ways [6]. A digital 2021 global report showed that there were more than 4.62 billion social media users worldwide in January 2022, equating to 58.4 percent of the total global population [7]. Moreover, a typical social media user actively uses an average of 7.5 different social platforms per month [7]. As a complement to interpersonal communication, social media makes individual information sharing behaviors complex, partly due to its specific characters. Stockmann and Luo classified Chinese cyberspace into one-way communication dominated by institutions, and interactive conversation between users [8]. They also found that Weibo was more like a public information exchange space, while WeChat served as an instant social interaction tool [8]. Chen proposed the definitions of public and non-public information sharing based on the visible range of information flow [9]. In other words, there exist different mechanisms of information diffusion among various social media platforms. During the COVID-19 pandemic, previous studies proved that social media have a crucial role in disseminating health information and tackling misinformation to form effective risk communication [10,11]. In China, Weibo is a primary online space for information sharing about risk situations. It is reported that there are 6.076 million pieces of COVID-19-related news shared by official institutions and media that aim to convey information and provide actual help [12]. In addition, the number of verified posts with the hashtag of seeking help from people suffering from diseases is 10,637 [12]. This information speeds up the process of risk management, which is beneficial for fighting the epidemic. It has been two years since the first COVID-19 case was discovered. Although China has entered a phase of regular prevention and control of COVID-19, Omicron as the globally dominant circulating variant still challenges China [13]. In the “New Normal” condition defined by the WHO, individual information sharing has been a prevention measure in daily life [14]. This makes it different from information behavior at the early emergency stage of COVID-19, and is a risk condition that refers not only to the regular schedule in the whole country, but also to sudden situations in local areas. Prior studies have only examined information sharing as a whole or a part of risk communication to explore the influencing factors in emergencies such as natural or man-made disasters [15,16]. External factors and internal factors, including risk situations [17], information environments [18], and personal factors [19] have impacts on emergency-induced information sharing, whereas the subdivision of information sharing and long-term risk reaction context has received little research attention. On the one hand, sharing information publicly and privately should be explored separately due to their different effects on risk communication and management. For example, Chen proposed that a single concern on public information sharing may lead to ignorance of potential information flow between certain individuals and within small groups, which is the key to demonstrating the incubation phase of risk [20]. On the other hand, information sharing has been a part of a particular lifestyle with the coexistence of the COVID-19. It is both an instant response and a habitual behavior with the appearance of the Omicron. Thus, it is necessary to reassess and complement the mechanisms of behaviors.

This study aims to examine the mechanism of information sharing intention, including subjective factors and the effect of news frame. Compared with other relevant studies, this study extends the understanding of information sharing in the “New Normal” of COVID-19. The followings are key concepts in this study. First, the study considers the different types of information sharing [9]. Public information sharing refers to information that can be viewed by a large online audience, while private information sharing is that in which people control the visible range of information transmitted according to subjective wishes and the interpersonal relationships [9]. Second, habit and the cognitive mechanism represented by four outcome expectations are the determinants of the model, and their relationship is explored. Habit is a specific mindset in a given context. Outcome expectations come from systematic thinking and refer to the anticipated consequences of intentional actions. Third, an information feature is used to identify groups, containing two news frames aimed at guiding actions and bringing comfort.

## 2. Literature Review

### 2.1. Information Sharing

Information sharing refers to the act of passing information from one person to another [21]. It is a type of information behavior that simultaneously satisfies senders’ and receivers’ needs. From the transmission point of view, the collective feature of this behavior is stressed. Bao and Bouthillier systematically explored the theoretical foundations of information sharing and defined it as a community-based collaborative behavior driven by mutual benefits [21]. Likewise, Rafaeli and Raban pointed out that information sharing provided helpful answers to requests for information [22]. In terms of the information sharing form, it was defined initially as news diffusion in interpersonal communication [23]. With the development of the internet, information sharing has become prevalent in online contexts such as social media platforms [22]. Under risk circumstances, information sharing generally refers to information exchange on emergencies between professionals, official organizations, and the public at risk. It has specific characteristics. For one thing, interpersonal communication such as information sharing shows protectiveness, which plays a role in personal understanding and acting upon critical situations [24]. For example, Chen defined information sharing in food safety issues as a personal protective act [9]. For another thing, social media has proved helpful because it can distribute risk-related information in real-time at a large scale [25]. This indicates that people can share information and thoughts concerning a risk event. These expressions result in free public discussions around issues, which is meaningful to note in risk communication and management.

The impact of information sharing at the individual and social levels has been revealed. For an individual, information sharing has impacts on cognition, emotion, and behavior. A study conducted by Ibrahim et al. demonstrated that information sharing could be a way of obtaining new information and comfort [26]. Moreover, Lane and Dal Cin proved that publicly sharing a selected video on one’s Facebook wall can increase willingness to volunteer for offline issue-related activities [27]. These findings are consistent with the studies of Myrick, which suggested that having shared information with others predicted prosocial cancer-related behaviors [28]. To the whole society, information sharing can contribute to risk communication and management. Liao et al. found that risk information sharing can be framed as economically beneficial and reduce risks in a community, both in the short and long term [16]. In addition, information sharing can establish relationships among individuals, groups, and agencies, which is the premise of collective action and risk management when facing emergencies [29].

Previous studies have taken information sharing types into consideration to demonstrate the behavioral mechanism. Generally, scholars distinguish information sharing based on the platform and the receiver’s characteristics. Some researchers investigated the role of platforms and indicated that people can control information sharing ranges through specific privacy settings. This suggests that people can determine information sharing behaviors based on the platform’s characteristics [30,31]. For example, Dong et al. identified that people’s health information sharing preferences were different on WeChat and Weibo due to the imitate degrees of the targeted audience [32]. Beyond platforms, Dubois et al. classified the behaviors from interpersonal closeness to illustrate that the same information may be shared with different groups [33]. Therefore, the critical point of information sharing is whether people control the visible range of transmitted information according to their subjective wishes. Chen claimed that information sharing can be divided into public and non-public information sharing [9]. Compared with observed and tracked public information sharing, private information sharing mainly consists of people’s instant responses and serves as a tool to strengthen social interaction. At the beginning stage of issues, people prefer to share information privately with friends one-to-one or within a group chat, contributing to incubating the risk [8]. This is different from public information sharing that usually facilitates the rise of online public opinion. In addition, the emotion function is more obvious when people share information privately. For example, conversations on WeChat can bring information, hedonic and social gratification [34]. However, it is difficult to consider private information sharing when managing risk, due to people’s control over information passage. That is why it lacks relevant exploration. The present study refers to the definition provided by Chen to discuss public sharing and private sharing intention separately [9]. Public information sharing intention refers to information that can be viewed by a large online audience, while the intention of private information sharing is so that people control the visible range of information transmitted according to their subjective wishes and the interpersonal relationships [9].

### 2.2. Factors Influencing Information Sharing

Under risk conditions, both objective and subjective factors have an influence on people’s perceptions and behavior. Systematic examinations about risk communication generally include contextual situations, stakeholders, and communication effectiveness [35]. A similar study conducted by Lindell and Perry supported this view [36]. They modified the protective action decision model and identified three core components, i.e., environmental and social cues, stakeholders’ perceptions, and actions. In the present study, we propose that information sharing was the individual subjective protective decision involved in the COVID-19 pandemic environment and online social media information. Hence, we discuss three aspects of influencing mechanism: risk characteristics as situational factors, information features as social cues, and subjective factors.

#### 2.2.1. Risk Characteristics

Considering the characteristics of risk that imply situational factors is necessary for risk communication. At the beginning of the COVID-19 pandemic, it was perceived as a dread risk with high uncertainty due to its novelty and unknown origins [37]. Social media, an outbreak communication platform, provided health information at this stage. Personal information exchange was prevalent at the outbreak of COVID-19. For example, the detected top behavior from personal Weibo account posts was sharing knowledge or information [38]. Besides, the level of risk is also related to personal perceptions and behaviors. Chen et al. found that people at the epicenter of the COVID-19 pandemic showed high information needs and kept seeking information to understand the situation [39]. Nevertheless, COVID-19 has created a condition that differs from other emergencies based on its extensive time period and constant evolution. The present condition is defined as the “New Normal” by the WHO [14]. It refers to the changes of a particular lifestyle, such as accepting daily prevention measures. Similarly, Li and Zhang described the present Chinese situation as a risk society with regular epidemic prevention and control, which indicates that the systematic way of dealing with hazards has formed at the society level and individual level [40].

Based on situational theory, there are multiple publics in response to various disaster predictions instead of a single, general public response [17]. How people view COVID-19 is related to the extent of pandemic exposure and perception. For people who live in high-risk areas or have direct risk experience, their perception is generally highly responsive. Their information sharing can be seen as a reaction to a sudden stimulation. People who only perceive risk from interpersonal and media information are close to the “New Normal”. They are accustomed to the COVID-19 physically and mentally. For this group, information sharing can be a kind of prevention measure in daily life. The critical difference between the two groups is the psychological distance from COVID-19. Referring to dimensions of the psychological distance proposed by Blauza et al., the geographical distance and the hypothetical distance have a relationship with people’s cognitive attitudes toward COVID-19 [41]. In addition, the direct physical and emotional experience caused by risks have an influence on individual risk perception [42]. The present study tries to explore the information sharing mechanism under a constant risk environment. Therefore, people who have been, or were in high-level risk residence, without vaccination, and with infected experience are not appropriate subjects. We focused on the group without direct experience of the COVID-19.

#### 2.2.2. Information Features

Information features conceptualized as structures, styles, and contents have also been proven to be relevant to information sharing intention [18]. Naveed et al. proposed high-level and low-level content-based features to analyze the likelihood of a retweet [43]. Low-level features contain words, emoticons, and on. High-level features refer to topics and sentiments. The news frames that can reflect the topic as a part of high-level content feature have been investigated in information sharing [44,45,46]. A systematic review summarized 15 general news frames and identified their prevalent usage in news reporting of health risks [47]. Liu and Xie showed that the health risk reporting frame, a content feature, has an influence on the users’ reposting behaviors about COVID-19 [48]. For example, economic, action, and reassurance frames were positively related to increased information sharing. In addition, researchers provided evidence that severity, efficacy, and emotionally evocative information can result in individuals presenting a higher sharing tendency [49,50].

The COVID-19 epidemic has been influencing people’s lives worldwide. Especially with the Omicron variant detected in Chinese some areas from December 2021, the government and public have shown concerns about this risk situation. At the initial stage of a disease outbreak, the media plays an essential role in providing information to the public [51]. Uribe concluded that early coverage focused on the disease situation rather than personal stories [52]. In China’s pre-crisis stage, Liu and Xie found that the top three news frames used on social media were the consequence frame that conveyed disease information (56.2%), the reassurance frame that brought hope for public (34.9%), and the action frame that promoted effective prevention measures (15%) [48]. When Omicron was discovered in China, there were regular strategies to prevent the spread of COVID-19. As a part of COVID-19, Omicron’s consequences are not the focus of the public due to the popularization of relevant knowledge. Therefore, the present study adopted the widely used action frame and the reassurance frame without regard to the consequences frame to discuss the following hypotheses:

**Hypothesis** **1a** **(H1a):** *There are differences in public information sharing intention between people exposed to reassurance-framed news and people exposed action-framed news.*

**Hypothesis** **1b** **(H1b):** *There are differences in private information sharing intention between people exposed to reassurance-framed news and people exposed action-framed news*.

#### 2.2.3. Subjective Factors

Subjective factors have been associated with behavior mechanisms as the core determinant. Some researchers have considered the cognitive and emotional responses aroused by risks. Researchers have suggested that information sharing was a behavior motivated by conscious subjective factors under specific situations [19,53,54]. Specifically, Yang et al. developed the Risk Information Seeking and Processing Model (RISP Model) to explain information sharing and confirmed that epistemic motivations and negative emotions can predict information sharing behaviors related to climate change [55]. These results are consistent with the research of Ruggiero, which assumed that people were aware of their needs and made rational decisions about information consumption based on the Uses and Gratifications Theory [56]. Recently, Xia et al. found that perceived benefits and costs were significantly associated with sharing intention on the basis of Social Exchange Theory [57]. Other researchers have highlighted the significance of unconscious factors in explaining usage behavior in the information system area [58,59].

As an unconscious factor, habit was a vital predicator of usage intention in information systems [60]. Anderson and Rainie pointed out that online information sharing will be a lifetime habit among millennials [61]. Additionally, context directly determined habitual behaviors [62]. The COVID-19 epidemic, as a public crisis, disrupted people’s daily routines to a new context that can result in the formation of new habits. In other words, two different sets of processes exist in information behaviors due to the types of subjective factors, which is consistent with Dual System Theory [63]. One system refers to non-conscious behavior without personal control, such as habit-induced information sharing. The other system denotes conscious behavior with systematic thinking, similar to information sharing, through the cognitive mechanism. Therefore, two paths to information sharing can be set up.

Relevant studies have also shown the relationship between cognitive mechanism and habit to support the association between two information sharing intention paths. The study conducted by Hsiao et al. showed that mediation effects of habit did occur between perceived usefulness and intention to information systems continue use [64]. Broersma and Swart found that people with high perceived value about COVID-19 information tend to form the novelty news usage habit and become frequent users [65].

Based on the above discussion, we proposed a research model. The model mainly focuses on subjective factors to explore the individual information sharing intention mechanism. It is assumed that cognitive mechanism and habit are the key factors that determine information sharing intention. These have an influence on public and private information sharing intention through two separate paths; such assumptions are plausible under the situation of “New Normal”. For people without direct high-risk experience, sharing the COVID-19 related information has become habitual behavior. When it comes to the threat brought by the Omicron variant, people may respond promptly including information sharing motived by the cognitional process to deal with the new risk. The cognitive mechanism is positively related to habit, which indicates that increasing systematic processing can lead to a heuristic system. Constant risk situations result in people accumulating cognitions, which are gradually transformed into a certain behavioral pattern such as a habit.

### 2.3. Habit

Habits are context-behavioral associations in memory and specific mindsets that grow as people experience rewards for a given action in a given context. Concerning the habit’s effect on an individual’s and behavior, Verplanken et al. demonstrated that a habit may profoundly affect information search, acquisition, and decision-making [66]. Hu et al. proved that habit formation influenced future social media usage behavior [67]. Specifically, Liu et al. found that the media usage habit affected public routed relevant information seeking [68]. In information sharing behaviors, Kim et al. found that sports fans who have already formed the habit of information sharing through hashtag usage tend to carry on with the behavior [69]. The present study added habit as an independent variable that has an influence on the intention of public and private information sharing.

**Hypothesis** **2a** **(H2a):** *Habit has significant positive effects on public information sharing intention.*

**Hypothesis** **2b** **(H2b):** *Habit has significant positive effects on private information sharing intention*.

When people are exposed to news, either sharing consequences or information processing choices can be partly predicted by the news frame. Alvi and Saraswat found that differences in the news characters’ effect result in systematic and heuristic processing choices [70]. Similarly, Nan et al. combined news frames with information processing mode and showed differences in groups and effects [71]. Combining existing evidence of the news frame’s effect and the above assumption that news frames impact information sharing, we propose that habit, as part of the information processing system, is influenced by news frames. Therefore, the following hypotheses were proposed:

**Hypothesis** **3a** **(H3a):** *There are differences in the impacts of habit on public information sharing intention between people exposed to reassurance-framed news and people exposed action-framed news*.

**Hypothesis** **3b** **(H3b):** *There are differences in the impacts of habit on private information sharing intention between people exposed to reassurance-framed news and people exposed action-framed news*.

### 2.4. Cognitive Mechanism

Cognitive mechanism is a core part of the research model that contributes to new habit formation after a sudden risk. Furthermore, it can predict the conscious part of individual behaviors, including information sharing. To explain the cognitive mechanism, social cognitive theory (SCT) emphasizes cognition’s primary role [72]. From an agentic perspective, Bandura stated that individuals are self-developing, self-regulating, self-reflecting, and proactive, instead of being just shaped by environments or inner forces [19]. Among referred factors in social cognitive theory(SCT), outcome expectation is central [73,74]. Outcome expectations are the anticipated consequences of intentional actions individuals choose to engage in, which partly come from systematic thinking [19]. The subcategories of outcome expectations have been discussed in knowledge sharing, health information sharing, and other issues-related information sharing. Outcome expectations can be distinguished by beneficiaries. For example, Wu et al. divided knowledge sharing outcome expectations in virtual communities into personal and community-related expectations [75]. In contrast, the community’s outcome expectation shows more public concern. In risk events, reducing the probability and severity of risk is the ultimate expected outcome. To protect oneself, primary outcome expectations are obtaining information and emotional support in the process of information sharing [26,54]. Out of reducing risk for others or the whole society, outcome expectations can be divided into benefiting others and contributing to risk management by public engagement [23,76]. Based on existing evidence, we defined four types of outcome expectations: (1) an information seeking outcome expectation that satisfies individual information needs; (2) an emotion regulation outcome expectation that provides individual emotion support; (3) an altruism outcome expectation that helps others in risk; (4) a public engagement expectation that engages risk management.

It has been proven that outcome expectations cause habits to some degree. Verplanken and Wood found that habit formation was steered by the cognitive process of comparing performance expectations about behavior with the actual outcome of the behavior [77]. Similarly, Hu et al. identified that expectation–confirmation of social media use was positively associated with habit [67]. Together with the above evidence, it is plausible that outcome expectation can be seen as a part of cognitive mechanism that has an impact on habit. With components of outcome expectations in this present study, we propose the following hypotheses:

**Hypothesis** **4a** **(H4a):** *Information seeking outcome expectation has significant positive effects on habit*.

**Hypothesis** **4b** **(H4b):** *Emotion regulation outcome expectation has significant positive effects on habit*.

**Hypothesis** **4c** **(H4c):** *Altruism outcome expectation has significant positive effects on habit.*

**Hypothesis** **4d** **(H4d):** *Public engagement outcome expectation has significant positive effects on habit*.

Previous studies have revealed the relationship between outcome expectations and information sharing upon social cognitive theory (SCT). Lin et al. identified that outcome expectations can improve users’ actual information sharing behavior on social media [78]. Under risk situations, a study by Chen found that outcome expectations can influence risk information sharing about food safety issues on social media [9]. Therefore, we inferred that every element of outcome expectancies may influence information sharing directly. Based on the classification of information sharing intention, the following subsections discuss and form relevant hypotheses.

#### 2.4.1. Information Seeking Outcome Expectation

Information seeking is the process or activity of attempting to obtain information [79]. In risk events, the RISP model (Risk Information Seeking Process) demonstrates that information processing behaviors such as information seeking and sharing are motivated by information need [80]. Satisfying information need is the information-seeking outcome expectation. During the COVID-19 epidemic, information seeking prompted by the uncertainty and lack of information may also increase social media users’ likelihood of sharing the information [81]. Thus, the following hypotheses were derived:

**Hypothesis** **5a** **(H5a):** *Information seeking outcome expectation has significant positive effects on public information sharing intention.*

**Hypothesis** **5b** **(H5b):** *Information seeking outcome expectation has significant positive effects on private information sharing intention*.

#### 2.4.2. Emotion Regulation Outcome Expectation

Outcomes of emotion regulation include better psychological health, increased well-being, and better coping with stressful life events [82]. Although there was no robust evidence that emotion regulation outcome expectations affect information sharing, prior studies have confirmed that sharing behaviors can contribute to obtaining the above emotion regulation outcomes. Sharing COVID-19 information might positively affect the general public’s psychological well-being [83]. Bazarova et al. also suggested that people derived intrinsic value and managed emotions by sharing personal information with others on social media [84]. When facing risk events, the expectation of regulating fear, anxiety, and other negative emotions aroused by the uncertainty of situations may cause sharing behaviors. Therefore, the following hypotheses were proposed:

**Hypothesis** **6a** **(H6a):** *Emotion regulation outcome expectation has significant positive effects on public information sharing intention.*

**Hypothesis** **6b** **(H6b):** *Emotion regulation outcome expectation has significant positive effects on private information sharing intention.*

#### 2.4.3. Altruism Outcome Expectation

Altruism is defined by Hare as self-destructive behavior performed for the benefit of others [85]. The outcome is to ultimately improve other’s lives, which includes an expectation on improving other’s lives [86]. Researchers have shown that knowledge sharing behaviors are motivated by altruism [75,87]. Meanwhile, Feinberg et al. strengthened the prosocial character of information sharing and showed that sharing negative evaluative information was a way to protect others from antisocial or exploitative behavior [88]. Hence, we hypothesized that:

**Hypothesis** **7a** **(H7a):** *Altruism outcome expectation has significant positive effects on public information sharing intention.*

**Hypothesis** **7b** **(H7b):** *Altruism outcome expectation has significant positive effects on private information sharing intention.*

#### 2.4.4. Public Engagement Outcome Expectation

Public engagement is utilitarian, civic-mindedness, and inspiration, which is important in promoting risk communication to reduce public risk [89]. Successful public engagement is critical for risk management and mitigation at individual and society levels [90]. Shah et al. have shown that people shared crisis information on SNSs to realize public engagement, which indicates that people can participate in a specific discussion via sharing crisis information [91]. According to the evidence that information sharing can serve as a way of public engagement, the following hypotheses are proposed:

**Hypothesis** **8a** **(H8a):** *Public engagement outcome expectation has significant positive effects on public information sharing intention.*

**Hypothesis** **8b** **(H8b):** *Public engagement outcome expectation has significant positive effects on private information sharing intention*.

### 2.5. Demographic Influences on Information Sharing

Prior research has indicated that some demographic variables can affect online information sharing attitudes and behaviors. Gender and education level has an effect but scholars have reached controversial conclusions. Indeed, gender has been tested as an influential variable in predicting information sharing cognitions, behaviors, and mechanisms. Different types of shared information and determinants behind it are significantly different between males and females [92]. Furthermore, Sun et al. examined how gender played a role in information sharing mechanisms and found that perceived benefit had a stronger effect on information sharing intention for men compared with women [93]. By contrast, Rousseau and Puttaraju found that there was no significant difference in males and females on the usage of SNSs for sharing their hobby or passion [94]. This result is consistent with the research of Malcolm [95]. In terms of education level, Rowley et al. proposed that having a higher level of educational attainment was associated with more frequent usage of the internet, including sharing information [96]. A study on cancer patients also showed that information sharing was higher among patients with more education [97].

Hence, we took gender and education level into consideration to ensure that the relationships between subjective factors and information sharing intention were not confounded.

## 3. Methods

### 3.1. Design

Based on a cross-sectional design, this study explored the mechanism of information sharing intention under regular COVID-19 prevention. Among the reasons for selecting mainland China instead of the whole of China is that mainland China adopts regular prevention strategies to deal with the dynamic epidemic, which differs from Chinese other regions [98,99]. Meanwhile, Weibo is one of widely used social media platforms in mainland China so that conducting the study only in mainland China is suitable [100]. We divided respondents into two groups based on the character of news they were exposed to (i.e., reassurance-framed news vs. action-framed news) and examined the difference in information sharing intention between them. The research was approved and managed by the Academic Ethics Committee.

### 3.2. Material

An online questionnaire was used to gather data in this study. The questionnaire was separated into three sections (Supplementary Materials S1). The first part of the questionnaire contained a news article as the stimulus and relevant items measuring the information sharing behaviors of the respondents. The stimulus materials contained two versions of news that focused on the situation of the Omicron variant in mainland China, which were different in the application of the news frame. We drafted news articles based on reports from Chinese official media on social media in the early stage of the Omicron’s spreading, mainly including news posted by People’s Daily and CCTV News from 14 January 2022 to 24 January 2022 on Weibo. These materials differed in how the headlines and main text were phrased, so that reassurance and action frames were constructed. To strengthen the credibility of news and simulate the true news reading experience, the presentation of news materials was designed as screenshots from social media with hidden sources (see the Supplementary Materials S1 for details). The manipulation of the news materials aimed to identify the focus of research, and following items that respondents were asked to answer were relevant to the information sharing behavior on the above specific-framed news.

The second section was the measurement of seven variables: Public Information Sharing Intention (PBSI), Private Information Sharing Intention (PRSI), Habit (HB), Information Seeking Outcome Expectation (IS), Emotion Regulation Outcome Expectation (ER), Altruism Outcome Expectation (AL), and Public Engagement Outcome Expectation (PE). The measurement items and sources are shown in Table 1.

The third part was related to variables of respondents. Considering the key subject of research, the items included demographic variables (such as gender, age, education level, occupation, and so on) and the interpersonal risk situation (including vaccinations, the risk degree of residence, infection condition of the COVID-19, and so on). Therefore, we can select the research groups and eliminate the influence caused by the difference of risk situations (see the Supplementary Materials S1 for details). In addition, gender and education level were included as control variable based on the discussion in Section 2.5.

### 3.3. Data Collection

The questionnaire was distributed by Tencent Questionnaire, which is a widely used questionnaire platform in China [105]. Two groups of respondents were recruited randomly via the Tencent questionnaire sample database that contains over 1 million people with verified personal information. They were required to complete different questionnaires separately, which differed in stimulus materials. The sample of respondents who were requested to answer the questionnaire that included reassurance-framed news was named the Reassurance Group; the other sample of who were requested to answer the questionnaire included action-framed news was named the Action Group. Data collection was conducted on the China mainland from 23 January 2022 to 25 January 2022. This period was selected because the situation with Omicron in the mainland China changed rapidly. Therefore, a three-day data collection period was used to ensure the obtained cross-sectional data can reflect the attitude of respondents under a similar risk situation of pandemic prevention and control.

### 3.4. Data Analysis

SmartPLS 3.2.2 was employed for data analysis [106]. SmartPLS is a structural equation model statistical analysis software based on the PLS algorithm. The first reason for using PLS-SEM is that compared with covariance-based structural equation modeling (CBM-SEM), PLS-SEM is more suitable for the analysis of a complex model in which data are not normally distributed [107]. Moreover, SmartPLS 3.2.2 supports multi-group analysis (MGA) based on non-parametric tests, which facilitates the comparison of models between different groups. The numbers of respondents in the two groups were 398 and 382, respectively, which met the PLS-SEM requirements for sample size [108]. Data analysis involved the following main steps. At first, measurement model testing was used to assess the reliability and validity of the model, mainly via Cronbach’s alpha, composite reliability, average variance extracted (AVE), and Fornell-Larcker criterion. The second step was the measurement invariance of composite models (MICOM) test to detect measurement invariance and then determine whether the two sets of data can be mixed for structural model evaluation. According to Hair, the measurement invariance of composite models (MICOM) procedure involves three steps: (1) configural invariance, (2) compositional invariance, and (3) equality of composite mean values and variances [108]. If configural and compositional invariance (step 1 and step 2) are established, partial measurement invariance is confirmed, allowing the path coefficient estimates across the groups to be compared. In addition, if partial measurement invariance is confirmed and the composites have equal mean values and variances across the groups, full measurement invariance is confirmed, which supports pooled data analysis. The third step was to evaluate the structural model to test whether the hypothesis of the relationship between the variables was true. Evaluation metrics of path analysis are R^2^ (explained variance), f^2^ (effect size), Q^2^ (predictive relevance), standardized root mean square residual (SRMR), and the size and statistical significance of the structural path coefficients.

## 4. Results

### 4.1. Sample Characteristics

A total of 1939 questionnaires were administrated, and 1101 questionnaires were returned. The Reassurance Group consisted of 550 respondents, while Action Group consisted of 551 respondents. All respondents were from mainland China. To eliminate the possible effect of risk situation on respondents’ information behaviors, respondents in the high-risk situation were excluded. The non-chosen respondents included people in high-level risk residence, without vaccination, and with infection experience. In addition, respondents who never or rarely engaged in information sharing in self-report were removed. Therefore, the final effective sample for the Reassurance Group and Action Group was 398 and 382, respectively. The details of the respondents are shown in Table 2.

As shown in Table 2, the sample was dominated by females (74.10%). Over half of respondents had completed higher education. Considering the role of gender in information sharing, the disproportionate distribution of gender may be related to different information sharing intention. Education level has been explored as a crucial factor influencing the way people share information [96]. Against the sample character in this study, the education level of the majority was college, so that its impact in the whole model should be measured. Therefore, we controlled demographic variables of gender and education level. Two dummy variables were used to assess them. Additionally, more than 80% of respondents were concentrated between 20 and 31 years old, which didn’t differ much from the user’s age data reported by Weibo Data Center [12]. As other researchers found, SNS users in China were generally young groups with ability and willingness [109]. Thus, sampling bias due to age is not a problem in the present study.

### 4.2. Measurement Models

At first, we assessed the structural model involving seven constructs and found that there were collinearity issues caused by the ER4 item and PBSI4 item. Therefore, we eliminated two items to examine the final measurement model. The evaluation of results was under the guidance of Hair [108]. Generally, reliability and internal consistency are verified by outer loading, Cronbach’s α, and composite reliability (CR). The loadings should be above the common threshold of 0.70 to ensure reliability. Cronbach’s α refers to all index variables having the same reliability, estimated based on the correlation between observed indicators. The acceptable Cronbach’s α value is between 0.70 and 0.80, and the excellent value is greater than 0.80. CR takes into account the difference between the outer loadings of index variables, which is comprehensively used to assess the consistency reliability of the measurement model. A CR that is higher than 0.70 can be accepted. In terms of validity, it is evaluated via convergent validity and discriminant validity. Convergent validity reflects the similarity of the results when different measurement methods are used to measure the same dimension. The average variance extracted (AVE) is used as the standard, and it must not be less than 0.50, which indicates that the construct can explain over half of the variance of index variables [110]. The Fornell-Larcker criterion assesses discriminant validity by comparing the square root of the AVE values with the latent variable correlations. When considering Fornell-Larcker, the square root of each construct’s AVE should be greater than its highest correlation with any other construct.

It can be seen from Table 3 that the loadings, the Cronbach’s α, and CR values in data from two groups were greater than 0.70, most of which were over 0.80 and approached an excellent standard. Based on these results, it can be concluded that these constructs had good reliability and internal consistency. The AVE values of all constructs in the model were above 0.5, showing good convergence validity. The results of the Fornell-Larcker analysis are presented in Table 4 and Table 5. The results met requirements so that discriminant validity was verified.

### 4.3. Measurement Model Invariance

Researchers suggest that the measurement invariance of composite models (MICOM) procedure in SmartPLS 3.3.2 can be employed to assess measurement invariance [108]. The MICOM is a three-step approach that includes configural invariance, compositional invariance, and equal means and variances. Suppose configural and compositional invariance (step 1 and step 2) are established without equal means and variances (step 3). In that case, partial measurement invariance is confirmed, which permits comparing the path coefficient estimates across the groups. When partial measurement invariance is established, and equal means and variances are obtained, full measurement invariance is established. MICOM results in Table 6 suggest that the two groups of data establish full measurement invariance. According to Hair, multigroup analysis (MGA) is used to assess whether the difference in path coefficients based on two different groups is statistically significant [108]. It can be seen from Table 7 that there was no significant difference in all path coefficients between two groups. Hence, H1a, H1b, H3a, and H3b were rejected. Two groups of data can be pooled to analyze to improve the statistical validity.

### 4.4. Structural Models

**Figure 1:**
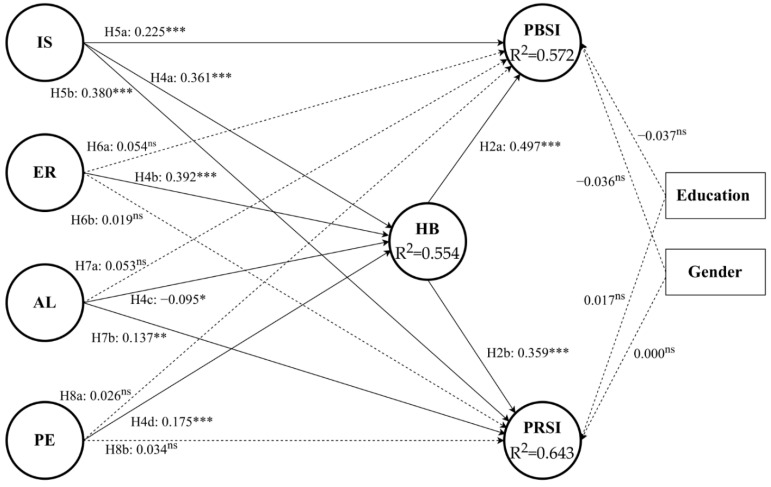
Results of the structural model analysis. Notes: * *p* < 0.05, ** *p* < 0.01, *** *p* < 0.001, ns = not significant.

Due to the establishment of full measurement invariance, we assessed structural models with the pooled data. At first, variance inflation factor (VIF) criteria in every construct were used to examine the potential collinearity within the structural model. If the value of VIF is less than 5, it can be proved that there are no collinearity issues. The highest value of the construct’s VIF was 3.003, which indicates all constructs were the absent concern of collinearity issues. Secondly, bootstrapping (5000 subsamples) was performed to examine the significance of path coefficients. The results in Table 8 and Figure 1 showed that altruism, emotion regulation and public engagement outcome expectations had no significant impact on public information sharing intention. Meanwhile, public engagement and emotion regulation outcome expectations had no significant impact on private information sharing intention. Except for the above five paths, other paths were significant. Therefore, H6a, H6b, H7a, H8a, and H8b were rejected while other hypotheses were supported. Regarding the demographic control variables, gender and education were not significantly associated with public and private information sharing intention. At the same time, it was shown that all the mediating effects are significant (see Table 9).

Then, we evaluated the predictive power of the model. In this process, R^2^, f^2^, and Q^2^ are three important indicators. R^2^ is the squared correlation of actual and predicted values, which represents a measure of in-sample predictive power. R^2^ values of 0.75, 0.50, or 0.25 for endogenous latent variables can be respectively described as substantial, moderate, or weak [111]. The results showed that the research model moderately demonstrated the variations. The f^2^ effect size enables analysis of the relevance of constructs in explaining selected endogenous constructs. Hair pointed out that f^2^ values of 0.02, 0.15, and 0.35 indicate an exogenous construct’s small, medium, or large effect, respectively, on an endogenous construct [108]. Based on results, medium effects exist in the two paths. One is the effect of emotion regulation outcome expectations on habit, the other refers to the impact of habit on public information sharing intention. Information seeking outcome expectations have a near medium impact on public and private information sharing intention and habit. Habit also has an almost medium impact on private information sharing intention. However, altruism and public engagement outcome expectations have almost no predictive power on public and private information sharing intention and habit. Similarly, emotion regulation outcome expectations also have almost no predictive power on public and private information sharing intention. We used a blindfolding procedure for assessing the predictive relevance (Q^2^ value) of the path model. If the Q^2^ value is above zero, the path model has predictive relevance for a selected reflective endogenous construct. In Table 8, the Q^2^ values of all constructs were above 0, which indicates a predictive relevance. Standardize Root Mean Square Residual (SRMR) is a model fit measure. In this study, the estimated SRMR value was 0.049, below the threshold value of 0.08. Therefore, the model had a good fit.

## 5. Discussion

Based on the above results, there was no significant difference in the information sharing intention between groups exposed to different news frames. To factors of information sharing, we found that outcome expectations and habits played a crucial role in public and private information sharing intention during the COVID-19 pandemic. The results showed that these factors had different impact mechanisms on information sharing, which included direct and indirect relationships. Notably, habit played a mediating role between outcome expectations and information sharing intention. The basic model established in this study was partially verified.

With respect to the role of news frames, the results showed no significant difference in information sharing mechanism between the two groups exposed to different frames. The result was inconsistent with studies of Liu and Xie [48]. This may be relevant to the new phase of the COVID-19. Generally, the need for information, including risk situations and prevention measures, was the key issue, especially when data on Coronavirus were changing daily [112]. However, in the “New Normal” phase of the COVID-19, people with a certain knowledge about the COVID-19 are prone to focus on the theme of news and neglect detailed information, which stresses the above discussion on routine protective information sharing. Information features may play a minimal role in the information sharing behaviors when people have formed specific information processing modes in a constant risk environment. Moreover, Banerjee and Meena suggested that there has been a hidden epidemic of “information” that includes repeated and detailed content about the virus, which leads to confusion [113]. Media organizations also seemed to create news inducing panic in an attempt to increase media consumption [114]. Compared with the threat of uncertainty, people are surrounded by massive information and familiar with possible news frames. The effect of the news frame is not strong, which shows the importance of the content value and credibility.

In terms of effect of outcome expectations and habit, this research found that their association with information sharing intention was supported. This shows two different processes exist in information sharing behaviors due to subjective factors. One refers that people’s expected outcomes form information sharing, which indicates that public and private information sharing are conscious and rational decisions in risk events through a systematic thinking process. These results are consistent with other studies, and suggest that subjective cognitive factors have an impact on individual information behavior [55,56]. The other is habit-induced information sharing. During a period with daily COVID-19 prevention, people who coexisted with long-term risk formed a routed system in the context-related information behavior. People are accustomed to sharing information as a habit to achieve the protective goal, which is similar to habitual information-seeking proposed by Griffin et al. [115]. In other words, results proved that information sharing was motivated by subjective factors, including habit, which was ignored in previous studies. In risk events that have features of emergencies and regular prevention, people not only share information as the instant response to risk out of certain outcome expectations but also take it as a habitual prevention measure into life.

However, different from two independent impacting paths of information sharing in previous studies, the results of this study verified the mediating role of habit in the model, which supplement the association of two approaches. Consistent with relevant findings, habit is formed by outcome expectations and has an impact on information sharing intention [69,77]. There is also an indirect relationship between outcome expectations and information sharing intention that is mediated by habit. Increasing the systematic process of evaluating outcomes can contribute to the heuristic process of habitual behavioral tendency, which further supports the view proposed by Chaiken and Ledgerwood [116]. Similar to evidence that heuristic processing can bias systematic processing, risk-context cues leads to people sharing information automatically, which can be seen as a result of systematic processing accumulations under the continuous exposure to risks [117]. When Omicron was discovered in the mainland China, it was a part of the COVID-19 pandemic. People who chronically live this New Normal situation without direct high-risk experience, may inherit systematic processing in the early stage of the COVID-19 and develop the stable habit of the Omicron-related information sharing, which is useful in improving understanding for information behavior.

This study showed that the four outcome expectations applied had different cognitive and habitual paths. Emotion regulation and public engagement outcome expectations only affected two types of information sharing intention through habit, without direct impact. In contrast, the impact of information seeking and altruism outcome expectations on sharing intention can be direct or indirect based on different types of information sharing.

For the emotion regulation outcome expectation, this affected public and private information sharing only through habit mediating. This result complemented the study of Yang et al., in which information sharing can be seen as an effective way of regulating emotion [83]. This may be related to the reality that people will share information privately and publicly only when people have received replies repeatedly to confirm their emotional expectation. Especially in risks, both sharing feelings with intimate relationships for particular support, and sharing information publicly to satisfy the need of expression, should be proved beneficial in the long term. This process results in the formation of habit. Likewise, public engagement outcome expectation impacts public and private information sharing intention mediated by habit and there is no direct association between public engagement outcome expectation and sharing intention. This finding differs from the findings of Shah et al. [91]. In other words, people who realize public engagement by sharing risk-related information show higher future share intention. This is the same in the public and private context. In risk situations, information sharing can’t be accepted as a way of public engagement instantly only after personal verifications in practice. To some extent, the outcome of public engagement cannot be observed quickly and directly, so that it needs more cognitive estimation and heuristic processes such as habit. That is why there is no direct effect between public engagement outcome expectations and information sharing intention.

In terms of the effect of information seeking and altruism outcome expectations, this study found that their mechanisms were more complex. There were two impacting approaches of information seeking outcome expectations on public and private information sharing intention. First, the finding provided more support for the research of Zhang and Cozma by classifying public and private information sharing [81]. People in mainland China have obtained basic knowledge and formed information-seeking habits since the outbreak of the epidemic. Still, emerging virus variants generate a need for new information to deal with the risks. This motivates people to improve knowledge levels by sharing information publicly and privately. Whether sharing behavior is public or private, both mechanisms include the cognitive system and habit-induced path. This is in accordance with the fact that people in risk situations satisfy information need as soon as possible in several kinds of ways. Concerning altruism outcome expectations, our results verified that they had both direct and indirect impacts on private information sharing intention while the direct impact on public information sharing intention was not significant. The two paths to private information sharing can be explained by people sharing information privately having different targeted audiences. Sharing with high-closeness groups approaches habit steered by altruism expectations and sharing with people suffering from risks in a certain period may be mainly influenced by habit. This finding can be supported by the naturally prosocial character of information sharing [88]. Altruism outcome expectations can also influence public sharing via habit. In this process, the direct impact of altruism on public sharing is not significant due to the uncertainty of the audience and specific needs. Only people who have formed a habit through repeating relevant cognitive process are prone to share information publicly.

Considering the role of gender and education, this study found that gender and education had no significant impact on two types of information sharing intention. A possible explanation is that subjective factors and mechanisms in the present study are common human intrinsic factors. This indicates that people show a relatively stable behavioral mode shaped by the “New Normal” of COVID-19, which gradually substitutes the effect of innate demographic characteristics.

Based on the above analysis, it is clear that both rational cognitive processing represented by outcome expectations, and heuristic processing such as habit, play an important role in public and private information sharing intention. More importantly, the two modes have associations that increasing outcome expectations will form a habit to influence information sharing. Therefore, it is particularly necessary to reconsider individual information behaviors under a specific risk situation that combines suddenness and routinization. The classification and mechanism of information sharing highlights the part of risk communication that is dominated by personal factors. This finding suggests that both public and private information sharing should be considered in risk management.

Several limitations to the present study need to be acknowledged. First, the study explored the mechanism of information sharing intention, which is not supported by empirical individual behavioral evidence. This is critical to make explanations more robust and better contribute to risk communication. Second, the risk situation was limited by selecting participants without direct high-risk experience to simulate reality. However, the intrinsic risk perception of people was not examined, which may cause minor errors. Meanwhile, the data of respondents showed an uneven distribution that needs further exploration on specific groups such as older groups. Therefore, more detailed research is needed, including actual sharing behaviors and classified groups.

## 6. Conclusions

This study set out to explore influencing factors and the mechanism of information sharing intention under risk conditions with suddenness and regularity in mainland China. News frames, the information feature, were included in the research to compare the possible differences they cause. After analyzing the data from 780 respondents in two groups, we found that reassurance and action frames had no impact on the mechanism of information sharing intention. This suggests that the role of news frames was not as significant as expected due to individual long-term risk exposure and habitual prevention. Outcome expectations and habit affected public and private information sharing intention while habit played a mediating role between outcome expectations and sharing intention. It was shown that both conscious cognitive factors and unconscious factors such as habit impact individual behavioral intention. There are two systems behind information sharing intention. In addition, the repeated cognitive process system can contribute to the formation of stable habits. The latter refers to a heuristic path. These findings suggest that the regularization of emergencies should be taken into consideration in risk communication among people who don’t have direct high-risk experience. A change of individual information behaviors brought by risks can be preserved as a habit, which deserves further exploration to better manage risk. More importantly, these findings demonstrate influencing factors and mechanisms in public and private information sharing. Private information sharing can be seen as a part of online risk incubation, which complements public information sharing. By classifying information sharing, this study expands the understanding of risk information flow.

We encourage future research in three directions. First, it is necessary to measure individual information sharing behaviors to explain the process more precisely, which can provide more robust evidence for this model. Second, as mentioned earlier, two news frames in this study had no significant impact on information sharing mechanism. However, the effect of news frames cannot be ignored in risk communication, so this needs more research in the different phases of risk situations. Finally, further research should examine the application possibilities of the information sharing intention model in classified risk situations and diversified groups.

## Figures and Tables

**Table 1:** Measurement of variables.

Construct	Question	Source
Public information sharing intention	PBSI1: When I go through the above type of COVID-19-related information on Weibo and other social media, I am willing to publicly share it on Weibo and other social media.	Chen [9]
	PBSI2: When I go through the above type of COVID-19-related information on Weibo and other social media, I am willing to I am willing to publicly share it twice or more on Weibo and other social media.	
	PBSI3: When I go through the above type of COVID-19-related information on Weibo and other social media, I am willing to publicly share it on multiple platforms.	
	PBSI4: When I go through the above type of COVID-19-related information on Weibo and other social media, I am willing to publicly share it on Weibo and other social media to make as many people see it as possible.	
Private information sharing intention	PRSI1: When I go through the above type of COVID-19-related information on Weibo and other social media, I am willing to share it with my friends.	Chen [9]
	PRSI2:When I go through the above type of COVID-19-related information on Weibo and other social media, I am willing to share it with others who are in a one-to-one chat with me.	
	PRSI3: When I go through the above type of COVID-19-related information on Weibo and other social media, I am willing to share it on platforms such as Moments that limits range of information flow.	
	PRSI4: When I go through the above type of COVID-19-related information on Weibo and other social media, I am willing to share it with familiar people including families, friends and so on.	
Habit	HB1: I always share the COVID-19-related information as a habit.	Limayem et al. [60] Liu & Li [101]
	HB2: Sharing the COVID-19-related information is natural to me.	
	HB3: Sharing the COVID-19-related information is automatic to me.	
	HB4: I often subconsciously share the COVID-19 information.	
Information seeking outcome expectation	IS1: When I share the COVID-19-related information, I want to obtain useful information from others’ feedback.	Chen et al. [20] Hilverda & Kuttschreuter [102]
	IS2: When I share the COVID-19-related information, other people will tell me what they know about these risks too.	
	IS3: When I share the COVID-19-related information, other people will exchange relevant information with me.	
	IS4: When I share the COVID-19-related information, I expect that other people share such information with me in the future.	
Emotion Regulation outcome expectation	ER1: Sharing the COVID-19-related information can alleviate my negative emotions.	Chen [9] Yu [103]
	ER2: Sharing the COVID-19-related information can bring a sense of relief to me.	
	ER3: Sharing the COVID-19-related information can make me feel positive.	
	ER4: Sharing the COVID-19-related information can help me regulate emotions.	
Altruism outcome expectation	AL1: Sharing the COVID-19-related information can warn others of risk.	Chen [9] Hennig-Thurau et al. [104]
	AL2: Sharing the COVID-19-related information can save others from risk.	
	AL3: Sharing the COVID-19-related information can keep others updated.	
	AL4: Sharing the COVID-19-related information can satisfy other’s interest.	
Public engagement outcome expectation	PE1: Sharing the COVID-19-related information can make it attract more attention.	Chen [9]
	PE2: Sharing the COVID-19-related information can contribute to more public discussion.	
	PE3: Sharing the COVID-19-related information can promote concern for public opinion and help to solve specific problems.	
	PE4: Sharing the COVID-19-related information can be an important way to express my opinion as a public.	

**Table 2:** Descriptive statistics of respondents’ demographics.

Variables		Reassurance Group		Action Group	
		*n* = 398		*n* = 382	
		*n*	%	*n*	%
Gender	Male	114	28.60	99	25.90
	Female	284	71.40	283	74.10
Age	20–31	354	88.90	338	88.48
	31–40	31	7.80	34	8.90
	41–50	9	2.30	6	1.57
	51–60	2	0.50	3	0.79
	61 or above	2	0.50	1	0.26
Education Level	High School or Less	73	18.30	57	14.92
	Undergraduate and Junior College	309	77.70	299	78.27
	Graduate Degree	16	4.00	26	6.81

**Table 3:** Results for the measurement models.

Constructs	Loadings		Cronbach’s α		CR		AVE	
	Reassurance Group	Action Group	Reassurance Group	Action Group	Reassurance Group	Action Group	Reassurance Group	Action Group
Altruism			0.904	0.891	0.933	0.925	0.776	0.754
AL1	0.893	0.882						
AL2	0.842	0.830						
AL3	0.908	0.904						
AL4	0.880	0.857						
Emotion regulation outcome expectation			0.911	0.895	0.944	0.934	0.848	0.826
ER1	0.890	0.902						
ER2	0.938	0.917						
ER3	0.934	0.908						
Habit			0.918	0.935	0.942	0.953	0.802	0.837
HB1	0.870	0.913						
HB2	0.918	0.912						
HB3	0.884	0.917						
HB4	0.909	0.917						
Information seeking outcome expectation			0.926	0.930	0.948	0.950	0.819	0.825
IS1	0.911	0.897						
IS2	0.904	0.911						
IS3	0.902	0.912						
IS4	0.902	0.915						
Public information sharing intention			0.931	0.914	0.956	0.946	0.878	0.853
PBSI1	0.927	0.913						
PBSI2	0.950	0.940						
PBSI3	0.935	0.917						
Public engagement outcome expectation			0.911	0.921	0.938	0.944	0.790	0.809
PE1	0.847	0.864						
PE2	0.923	0.908						
PE3	0.902	0.916						
PE4	0.882	0.908						
Private information sharing intention			0.894	0.886	0.927	0.922	0.760	0.747
PRS1	0.909	0.896						
PRSI2	0.906	0.884						
PRSI3	0.823	0.797						
PRSI4	0.846	0.878						

**Table 4:** Fornell-Larcker Criteria for the Reassurance Group.

	AL	ER	HB	IS	PBSI	PE	PRSI
AL	0.881						
ER	0.406	0.921					
HB	0.372	0.641	0.895				
IS	0.609	0.598	0.63	0.905			
PBSI	0.373	0.515	0.731	0.602	0.937		
PE	0.744	0.499	0.509	0.65	0.475	0.889	
PRSI	0.552	0.594	0.716	0.728	0.726	0.592	0.872

**Table 5:** Fornell-Larcker Criteria for the Action Group.

	AL	ER	HB	IS	PBSI	PE	PRSI
AL	0.869						
ER	0.467	0.909					
HB	0.514	0.686	0.915				
IS	0.698	0.613	0.689	0.909			
PBSI	0.558	0.601	0.708	0.677	0.923		
PE	0.761	0.574	0.625	0.76	0.599	0.899	
PRSI	0.608	0.533	0.685	0.754	0.75	0.648	0.864

**Table 6:** Results of invariance measurement testing.

Constructs	Configural Invariance	Compositional Invariance		Partial Measurement Invariance	Equal Mean Assessment			Equal Variance Assessment			Full Measurement Invariance
		Original Correlation	Confidence Interval		Difference	Confidence Interval	Equal	Difference	Confidence Interval	Equal	
AL	Yes	1.000	[0.999, 1.000]	Yes	0.127	[−0.142, 0.133]	Yes	−0.090	[−0.203, 0.211]	Yes	Yes
ER	Yes	1.000	[1.000, 1.000]	Yes	0.010	[−0.138, 0.146]	Yes	0.065	[−0.177, 0.168]	Yes	Yes
HB	Yes	1.000	[1.000, 1.000]	Yes	−0.015	[−0.139, 0.142]	Yes	0.001	[−0.172, 0.154]	Yes	Yes
IS	Yes	1.000	[1.000, 1.000]	Yes	−0.043	[−0.138, 0.143]	Yes	0.040	[−0.196, 0.207]	Yes	Yes
PBSI	Yes	1.000	[1.000, 1.000]	Yes	−0.009	[−0.142, 0.139]	Yes	0.057	[−0.169, 0.161]	Yes	Yes
PE	Yes	1.000	[1.000, 1.000]	Yes	0.072	[−0.141, 0.142]	Yes	−0.124	[−0.209, 0.208]	Yes	Yes
PRSI	Yes	1.000	[1.000, 1.000]	Yes	−0.057	[−0.144, 0.141]	Yes	0.048	[−0.173, 0.188]	Yes	Yes

**Table 7:** Results of MGA.

Hypothesis	Relationships	Path Coefficient Difference (Reassurance Group—Action Group)	Supported
H4c	AL → HB	−0.111 ^ns^	-
H7a	AL → PBSI	−0.138 ^ns^	-
H7b	AL → PRSI	0.038 ^ns^	-
H4b	ER → HB	−0.009 ^ns^	-
H6a	ER → PBSI	−0.132 ^ns^	-
H6b	ER → PRSI	0.106 ^ns^	-
H2a	HB → PBSI	0.195 ^ns^	No
H2b	HB → PRSI	0.077 ^ns^	No
H4a	IS → HB	0.015 ^ns^	-
H5a	IS → PBSI	−0.03 ^ns^	-
H5b	IS → PRSI	−0.116 ^ns^	-
H4d	PE → HB	0.029 ^ns^	-
H8a	PE → PBSI	0.042 ^ns^	-
H7b	PE → PRSI	−0.017 ^ns^	-

Notes: ns = not significant.

**Table 8:** Results for structural models.

Hypothesis	Relationships	Path Coefficients	Standard Deviation	T Statistics	Supported	R^2^	f^2^	Q^2^
H4c	AL → HB	−0.095 *	0.038	2.518	Yes	0.554	0.008	0.449
H4b	ER → HB	0.392 ***	0.039	10.153	Yes		0.211	
H4a	IS → HB	0.361 ***	0.04	9.019	Yes		0.118	
H4d	PE → HB	0.175 ***	0.043	4.031	Yes		0.023	
H7a	AL → PBSI	0.053 ^ns^	0.054	0.965	No	0.572	0.003	0.489
H6a	ER → PBSI	0.054 ^ns^	0.043	1.269	No		0.003	
H2a	HB → PBSI	0.497 ***	0.050	9.89	Yes		0.257	
H5a	IS → PBSI	0.225 ***	0.054	4.17	Yes		0.043	
H8a	PE → PBSI	0.026 ^ns^	0.059	0.441	No		0.001	
-	education → PBSI	−0.037 ^ns^	0.037	0.999	No		0.003	
-	gender → PBSI	−0.036 ^ns^	0.025	1.399	No		0.003	
H7b	AL → PRSI	0.137 **	0.044	3.081	Yes	0.643	0.021	0.479
H6b	ER → PRSI	0.019 ^ns^	0.038	0.499	No		0.001	
H2b	HB → PRSI	0.359 ***	0.042	8.566	Yes		0.161	
H5b	IS → PRSI	0.380 ***	0.050	7.529	Yes		0.146	
H8b	PE → PRSI	0.034 ^ns^	0.047	0.727	No		0.001	
-	education → PRSI	0.017 ^ns^	0.026	0.644	No		0.001	
-	gender → PRSI	0.000 ^ns^	0.022	0.018	No		0.000	

Notes: * *p* < 0.05, ** *p* < 0.01, *** *p* < 0.001, ns = not significant(*p* > 0.05).

**Table 9:** Results for indirect effects.

	Indirect Effects	Standard Deviation	T Statistics
AL → HB → PBSI	−0.047 *	0.020	2.371
ER → HB → PBSI	0.195 ***	0.027	7.187
IS → HB → PBSI	0.179 ***	0.028	6.359
PE → HB → PBSI	0.087 ***	0.023	3.728
AL → HB → PRSI	−0.034 *	0.015	2.340
ER → HB → PRSI	0.141 ***	0.021	6.561
IS → HB → PRSI	0.130 ***	0.021	6.139
PE → HB → PRSI	0.063 ***	0.018	3.584

Notes: * *p* < 0.05, *** *p* < 0.001.

## Data Availability

The data presented in this study can be provided upon request to the corresponding author. For ethical reasons, these data cannot be made public.

## Supplementary Materials

The following supporting information can be seen downloaded at: https://www.mdpi.com/article/10.3390/ijerph19095552/s1, Supplementary Materials S1: Materials and main items of the questionnaire.
