# Supplementary material for: Public and Private Information Sharing under “New Normal” of COVID-19: Understanding the Roles of Habit and Outcome Expectation
Source: Int J Environ Res Public Health. 2022 May 3;19(9):5552. doi: 10.3390/ijerph19095552 (PMC9105274; doi:10.3390/ijerph19095552)
Supplement: Supplementary file 1 [file ijerph-19-05552-s001.zip › ijerph-1650958-supplementary.pdf]

Supplementary material: Materials and main items of the questionnaire.

Part I

Stimulus material 1:

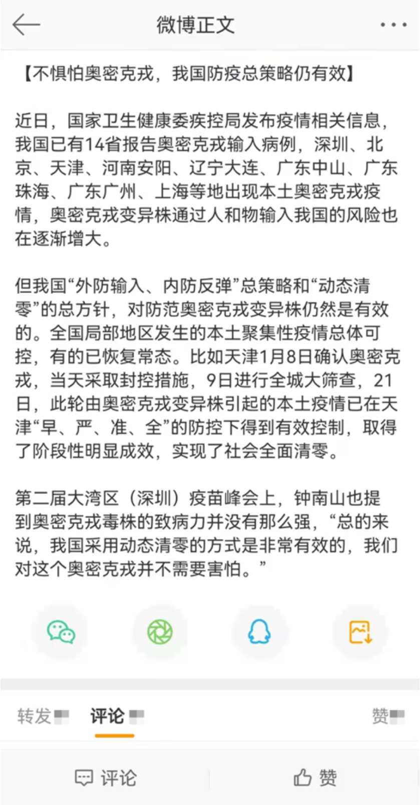

微博正文

【不惧怕奥密克戎，我国防疫总策略仍有效】

近日，国家卫生健康委疾控局发布疫情相关信息，我国已有14省报告奥密克戎输入病例，深圳、北京、天津、河南安阳、辽宁大连、广东中山、广东珠海、广东广州、上海等地出现本土奥密克戎疫情，奥密克戎变异株通过人和物输入我国的风险也在逐渐增大。

但我国“外防输入、内防反弹”总策略和“动态清零”的总方针，对防范奥密克戎变异株仍然是有效的。全国局部地区发生的本土聚集性疫情总体可控，有的已恢复常态。比如天津1月8日确认奥密克戎，当天采取封控措施，9日进行全城大筛查，21日，此轮由奥密克戎变异株引起的本土疫情已在天津“早、严、准、全”的防控下得到有效控制，取得了阶段性明显成效，实现了社会全面清零。

第二届大湾区（深圳）疫苗峰会上，钟南山也提到奥密克戎毒株的致病力并没有那么强，“总的来说，我国采用动态清零的方式是非常有效的，我们对这个奥密克戎并不需要害怕。”

转发 评论 赞

评论 赞

【Don't be afraid of the Omicron: Chinese regular prevention is still effective】

The National Health Commission of the People's Republic of China recently released relevant information on the COVID-19 epidemic. Data shows that fourteen provinces in the China mainland have reported imported cases of Omicron. The regions include Shenzhen, Beijing, Tianjin, Anyang in Henan, Dalian in Liaoning, Zhongshan in Guangdong, Zhuhai in Guangdong, Guangzhou in Guangdong, Shanghai, etc. With the emergence of the Omicron case, the risk of Omicron variants being imported into our country through people and things is gradually increasing.

However, Chinese regular prevention that consists of "preventing inbound cases and domestic resurgence" and "dynamic clearance" is still effective. The local clustered epidemics in regions are generally under control while some have returned to normal. For example, Tianjin confirmed Omicron on January 8, and took measures to block and control it on the same day. On the 9th, a city-wide screening was carried out. On the 21st, this round of local epidemic caused by the Omicron was controlled quickly and accurately, which has achieved part of results by completing clearance

At the 2nd Greater Bay Area (Shenzhen) Vaccine Summit, Zhong Nanshan also mentioned that the pathogenicity of the Omicron strain is not strong. He said, "In general, Chinese dynamic clearing method is very effective. We don't need to be afraid of this Omicron."

Stimulus material 2:

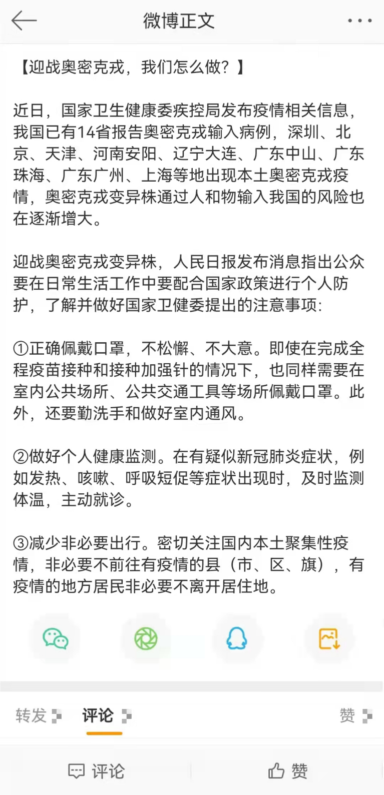
微博正文

【迎战奥密克戎，我们怎么做？】

近日，国家卫生健康委疾控局发布疫情相关信息，我国已有14省报告奥密克戎输入病例，深圳、北京、天津、河南安阳、辽宁大连、广东中山、广东珠海、广东广州、上海等地出现本土奥密克戎疫情，奥密克戎变异株通过人和物输入我国的风险也在逐渐增大。

迎战奥密克戎变异株，人民日报发布消息指出公众要在日常生活工作中要配合国家政策进行个人防护，了解并做好国家卫健委提出的注意事项：

①正确佩戴口罩，不松懈、不大意。即使在完成全程疫苗接种和接种加强针的情况下，也同样需要在室内公共场所、公共交通工具等场所佩戴口罩。此外，还要勤洗手和做好室内通风。

②做好个人健康监测。在有疑似新冠肺炎症状，例如发热、咳嗽、呼吸短促等症状出现时，及时监测体温，主动就诊。

③减少非必要出行。密切关注国内本土聚集性疫情，非必要不前往有疫情的县（市、区、旗），有疫情的地方居民非必要不离开居住地。

转发 评论 赞

评论 赞

【What can we do to fight against the Omicron?】

The National Health Commission of the People's Republic of China recently released relevant information on the COVID-19 epidemic. Data shows that fourteen provinces in the China mainland have reported imported cases of Omicron. The regions include Shenzhen, Beijing, Tianjin, Anyang in Henan, Dalian in Liaoning, Zhongshan in Guangdong, Zhuhai in Guangdong, Guangzhou in Guangdong, Shanghai, etc. With the emergence of the Omicron case, the risk of Omicron variants being imported into our country through people and things is gradually increasing.

In the battle against the Omicron, the People's Daily issued a message stating that the public should cooperate with national policies for personal protection in their daily work. Following the advice from the National Health Commission of the People's Republic of China is necessary.

①Wear the mask correctly, be not slack or careless. Even after completing vaccination and the booster shot, it is also necessary to wear masks in indoor public places, public transportation, and other places. In addition, wash your hands frequently and do good indoor ventilation.

② Monitor personal health status. If you discover the suspected symptoms of the COVID-19 , such as fever, cough, shortness of breath, and other symptoms, monitor the body temperature in time and seek medical treatment.

③ Reduce non-essential travel. Pay close attention to the local cluster epidemic in China, do not go to the regions with the epidemic unless necessary, and the residents of the place with the epidemic do not leave the place of residence unless necessary.

Part II

(A seven-point Likert scale was employed, with 1 corresponding to strongly disagree, 7 strongly agree.)

Public information sharing intention

- When I go through the above type of COVID-19-related information on Weibo and other social media, I am willing to publicly share it on Weibo and other social media.

☐	☐	☐	☐	☐	☐	☐
1	2	3	4	5	6	7

- When I go through the above type of COVID-19-related information on Weibo and other social media, I am willing to publicly share it twice or more on Weibo and other social media.

☐	☐	☐	☐	☐	☐	☐
1	2	3	4	5	6	7

- When I go through the above type of COVID-19-related information on Weibo and other social media, I am willing to publicly share it on multiple platforms.

☐	☐	☐	☐	☐	☐	☐
1	2	3	4	5	6	7

- When I go through the above type of COVID-19-related information on Weibo and other social media, I am willing to publicly share it on Weibo and other social media to make as many people see it as possible.

☐	☐	☐	☐	☐	☐	☐
1	2	3	4	5	6	7

Private information sharing intention

- When I go through the above type of COVID-19-related information on Weibo and other social media, I am willing to share it with my friends.

☐	☐	☐	☐	☐	☐	☐
1	2	3	4	5	6	7

- When I go through the above type of COVID-19-related information on Weibo and other social media, I am willing to share it with others who are in a one-to-one chat with me.

☐	☐	☐	☐	☐	☐	☐
1	2	3	4	5	6	7

- When I go through the above type of COVID-19-related information on Weibo and other social media, I am willing to share it on platforms such as Moments that limits range of information flow.

☐	☐	☐	☐	☐	☐	☐
1	2	3	4	5	6	7

- When I go through the above type of COVID-19-related information on Weibo and other social media, I am willing to share it with familiar people including families, friends and so on.

☐	☐	☐	☐	☐	☐	☐
1	2	3	4	5	6	7

Habit

- I always share the COVID-19-related information as a habit.

☐	☐	☐	☐	☐	☐	☐
1	2	3	4	5	6	7

- Sharing the COVID-19-related information is natural to me.

☐	☐	☐	☐	☐	☐	☐
1	2	3	4	5	6	7

- Sharing the COVID-19-related information is automatic to me.

☐	☐	☐	☐	☐	☐	☐
1	2	3	4	5	6	7

- I often subconsciously share the COVID-19 information.

☐	☐	☐	☐	☐	☐	☐
1	2	3	4	5	6	7

Information seeking outcome expectation

- When I share the COVID-19-related information, I want to obtain useful information from others' feedback.

☐	☐	☐	☐	☐	☐	☐
1	2	3	4	5	6	7

- When I share the COVID-19-related information, other people will tell me what they know about these risks too.

☐	☐	☐	☐	☐	☐	☐
1	2	3	4	5	6	7

- When I share the COVID-19-related information, other people will exchange relevant information with me.

☐	☐	☐	☐	☐	☐	☐
1	2	3	4	5	6	7

- When I share the COVID-19-related information, I expect that other people share such information with me in the future.

☐	☐	☐	☐	☐	☐	☐
1	2	3	4	5	6	7

Emotion regulation outcome expectation

- Sharing the COVID-19-related information can alleviate my negative emotions.

☐	☐	☐	☐	☐	☐	☐
1	2	3	4	5	6	7

- Sharing the COVID-19-related information can bring a sense of relief to me.

☐	☐	☐	☐	☐	☐	☐
1	2	3	4	5	6	7

- Sharing the COVID-19-related information can make me feel positive.

☐	☐	☐	☐	☐	☐	☐
1	2	3	4	5	6	7

- Sharing the COVID-19-related information can help me regulate emotions.

☐	☐	☐	☐	☐	☐	☐
1	2	3	4	5	6	7

Altruism outcome expectation

- Sharing the COVID-19-related information can warn others of risk.

☐	☐	☐	☐	☐	☐	☐
1	2	3	4	5	6	7

- Sharing the COVID-19-related information can save others from risk.

☐	☐	☐	☐	☐	☐	☐
1	2	3	4	5	6	7

- Sharing the COVID-19-related information can keep others updated.

☐	☐	☐	☐	☐	☐	☐
1	2	3	4	5	6	7

- Sharing the COVID-19-related information can satisfy other's interest.

☐	☐	☐	☐	☐	☐	☐
1	2	3	4	5	6	7

Public engagement outcome expectation

- Sharing the COVID-19-related information can make it attrack more attention.

☐	☐	☐	☐	☐	☐	☐
1	2	3	4	5	6	7

- Sharing the COVID-19-related information can contribute to more public discussion.

☐	☐	☐	☐	☐	☐	☐
1	2	3	4	5	6	7

- Sharing the COVID-19-related information can promote concern for public opinion and help to solve specific problems.

☐	☐	☐	☐	☐	☐	☐
1	2	3	4	5	6	7

- Sharing the COVID-19-related information can be an important way to express my opinion as a public.

☐	☐	☐	☐	☐	☐	☐
1	2	3	4	5	6	7

Part III

- Your gender: ______
- Your age: ______
- Your education level: ______
- Your disposable income per month: _______
- Your occupation: _______
- What is your condition of COVID-19 vaccinations?
  - ☐ Not vaccinated.
  - ☐ At least one shot vaccinated except the booster shot.
  - ☐ Vaccinated including the booster shot.
- Have you or people close to you ever been in high or medium risk regions?
  - ☐ Yes.
  - ☐ No.
- Have you or people close to you ever undergone quarantine and medical observation？
  - ☐ Yes.
  - ☐ No.
- Have you or people close to you ever been diagnosed with COVID-19?
  - ☐ Yes.
  - ☐ No.
